# Beneficial Effects of Slow-Release Large Neutral Amino Acids after a Phenylalanine Oral Load in Patients with Phenylketonuria

**DOI:** 10.3390/nu13114012

**Published:** 2021-11-10

**Authors:** Iris Scala, Daniela Concolino, Anna Nastasi, Giulia Esposito, Daniela Crisci, Simona Sestito, Stefania Ferraro, Lucia Albano, Margherita Ruoppolo, Giancarlo Parenti, Pietro Strisciuglio

**Affiliations:** 1 Department of Maternal and Child Health, Federico II University Hospital, 80131 Naples, Italy; 2 Pediatric Unit, Department of Health Sciences, Magna Graecia University of Catanzaro, 88100 Catanzaro, Italy; dconcolino@unicz.it (D.C.); sestitosimona@unicz.it (S.S.); ferrarostefania987@gmail.com (S.F.); 3 Physiology Nutrition Unit, Department of Clinical Medicine and Surgery, Federico II University, 80131 Naples, Italy; nastasi.annamaria@gmail.com; 4 Department of Translational Medical Science, Section of Pediatrics, Federico II University, 80131 Naples, Italy; giuliaesposito3107@gmail.com (G.E.); parenti@unina.it (G.P.); pietro.strisciuglio@unina.it (P.S.); 5 CEINGE Biotecnologie Avanzate Scarl, 80131 Naples, Italy; crisci@ceinge.unina.it (D.C.); albano@ceinge.unina.it (L.A.); margherita.ruoppolo@unina.it (M.R.); 6 Department of Molecular Medicine and Medical Biotechnologies, Federico II University, 80131 Naples, Italy; 7 Telethon Institute of Genetics and Medicine, 80078 Pozzuoli, Italy

**Keywords:** phenylketonuria, large neutral amino acids, phenylalanine, tyrosine, PKU, LNAA, srLNAA, Phe, Tyr, phenylalanine load

## Abstract

The mainstay of phenylketonuria treatment is a low protein diet, supplemented with phenylalanine (Phe)-free protein substitutes and micronutrients. Adhering to this diet is challenging, and even patients with good metabolic control who follow the dietary prescriptions in everyday life ignore the recommendations occasionally. The present study explores the ability of slow-release large neutral amino acids (srLNAAs) to prevent Phe increase following a Phe dietary load. Fourteen phenylketonuric patients aged ≥13 years were enrolled in a 6-week protocol. Oral acute Phe loads of 250 and 500 mg were added to the evening meal together with srLNAAs (0.5 gr/kg). Phe and tyrosine were dosed before dinner, 2h-after dinner, and after the overnight fast. After oral Phe loads, mean plasma Phe remained stable and below 600 µmol/L. No Phe peaks were registered. Tyrosine levels significantly increased, and Phe/Tyrosine ratio decreased. No adverse events were registered. In conclusion, a single oral administration of srLNAAs at the dose of 0.5 gr/kg is effective in maintaining stable plasma Phe during acute oral loads with Phe-containing food and may be added to the dietetic scheme in situations in which patients with generally good adherence to diet foresee a higher than prescribed Phe intake due to their commitments.

## 1. Introduction

Phenylketonuria (PKU; MIM #261600) is an inborn error of metabolism caused by the deficiency of phenylalanine hydroxylase (PAH, EC 1.14.16.1), the hepatic enzyme that converts phenylalanine (Phe) into tyrosine (Tyr), using tetrahydrobiopterin (BH4) as a coenzyme. PKU patients are diagnosed via neonatal screening and treated with a Phe-restricted diet to prevent neurological complications due to pathological Phe levels in the blood [1]. Diet for PKU is based on the restriction of protein-containing food in accordance with Phe tolerance, supplementation with protein substitutes (PS) and micronutrients, and the use of special low protein food. In patients with PKU, dietary therapy is recommended for life; indeed, patients with poor metabolic control may experience executive function impairment [2] and deficit of working memory [3,4], inhibitory control [3,5,6], sustained attention [7], cognitive flexibility [6,8], verbal fluency [4,9], and planning [7,10,11]. However, dietary treatment is hampered by psychological discomfort and reduced compliance to the diet after the first 4–5 years of life. Adherence to the diet decreases with increasing age [12]. Among PKU adult patients, more than half report low adherence to medical prescriptions, increased consumption of natural protein sources, and suboptimal use of PS [13]. From adolescence onwards, deviation from the diet often occurs when patients are out of the home, mainly because of embarrassment and fear of social isolation. Other factors hampering adherence to the diet include logistical problems in the workplace or while traveling, the low palatability of PS, and the lack of apparent correlation between well-being and metabolic control, as patients do not feel sick and do not perceive the disease as dangerous to their health [14]. Industries have developed a variety of differently flavored Phe-free amino acids mixtures (with or without micronutrients) to improve palatability, including glycomacropeptide-based PS derived from a whey-based low Phe natural protein and amino acid mixtures enriched with large neutral amino acids (LNAAs). LNAAs may have beneficial effects in PKU by taking advantage of the competitive inhibition of non-Phe LNAAs with Phe at the L-system transporters. Indeed, Phe and other neutral amino acids (LNAAs: tyrosine, tryptophan, threonine, methionine, valine, isoleucine, leucine, and histidine) share the same transporter in the brain and the intestinal mucosa and may reduce brain Phe levels, stabilize plasma Phe, and increase non-Phe LNAAs in the brain, such as Tyr and tryptophan, and the synthesis of dopamine and serotonin neurotransmitters [15,16]. In the intestine, LNAAs are transported both at the apical and at the basolateral membrane. At the apical membrane, the transport of LNAAs is almost entirely Na^+^-dependent through a transporter named system B^0^ (B0-AT1, SLC6A19) [17]. Always at the apical membrane, the ASCT2 transporter (SLC1A5) exerts an antiport of amino acids and, thus, does not contribute to the net transport of neutral amino acids across the apical cell surface [18]. At the intestinal epithelial basolateral membrane, LNAA transport occurs mainly through the L-system transporter LAT2 (SLC7A8) that forms a heterodimer with the 4F2 heavy chain (4F2hc). The LAT2-4F2hc complex shows the highest expression in jejunum and ileum [19]. While LAT1 is preferentially expressed in the brain, the brain-blood barrier, and the placenta, LAT2 is overwhelmingly expressed in the small intestine, kidney, lung, heart, and spleen [20,21]. It mediates a Na^+^-independent obligatory exchange of substrates with a 1:1 stoichiometry [22] with specificity toward LNAAs, including small ones [23], and thyroid hormones [24]. LAT2-4F2hc exhibits higher affinity (Km = 30–150 mM) to tyrosine, phenylalanine, tryptophan, threonine, asparagine, isoleucine, cysteine, serine, leucine, valine, and glutamine and relatively lower affinity (Km = 180–300 mM) to histidine, alanine, methionine, and glycine [25]; it has a higher affinity for L-Phe than LAT1. It is mainly involved in transepithelial transport rather than cellular accumulation. In the clinical setting, long-term treatments with PS enriched with LNAAs have shown benefits in PKU patients by improving distress and well-being rates, executive functions, attention, vigilance [26,27], treatment adherence, and quality of life [28]. In blood, long-term LNAA supplementation induces a significant increase of Tyr plasma levels and a consequent decrease of Phe/Tyr ratio [27,29] while Phe levels remain unchanged. Conversely, short-term treatments with LNAAs were proven to be effective in reducing plasma Phe levels in other studies [30,31,32]. Among LNAAs supplements, slow-release LNAAs (srLNAAs) are prolonged-release formulations that provide a physiological absorption of amino acids similar to natural proteins. This technology may be helpful in the optimal exploitation of the competitive inhibition of LNAAs with Phe derived from natural proteins and, putatively, prevent plasma Phe peaks. In addition, amino acids are incorporated into micro granules that are coated with a methylcellulose film to prevent any unpleasant taste, enhancing adherence to dietary prescriptions [28]. A recent Italian survey on the dietary habits of PKU patients highlighted that 80% of patients generally have lunch or dinner out of the home more than twice per week. Natural sources of protein were consumed frequently; usually, 1–3 times/week [13,28], with acute loads of Phe derived from foods not allowed in the diet. In real life, this is also true for a number of patients with generally good metabolic control who, however, admit to ignoring the recommendations on specific occasions, such as weekend dinners or parties, even though they follow the dietary prescriptions in everyday life. Focusing on these patients, the present study explores the ability of srLNAAs to prevent plasma Phe increase following an acute dietary Phe load.

## 2. Materials and Methods

### 2.1. Study Population

Twenty adolescent and adult patients with PKU requiring dietary treatment were enrolled at the Department of Maternal and Child Health, Federico II University Hospital, Naples, Italy, and at the Department of Pediatrics, University Magna Graecia, Catanzaro, Italy. Of these patients, 14 (10 females/4 males) correctly adhered to the study protocol and were included in the analysis. The mean age at enrolment was 22 years (range 14–33 years). Inclusion criteria were (i) diagnosis of PKU by newborn screening, requiring Phe-restricted diet and amino acids mixtures; (ii) age at enrolment ≥13 years old; (iii) adherence to the prescribed dietary regimen; (iv) adherence to the study protocol; (v) signature of informed consent by the patient or the legal representative. Exclusion criteria were (i) HPA; (ii) patients aged <13 years; (iii) pregnancy (presumed or ascertained); (iv) deviation from the study protocol, and (v) absence of informed consent signature. Patients’ demographics, phenotype, genotype, and the quantity (in grams) of srLNAAs load are described in Table 1. Table 1 also shows the amino acid intake from PS and natural proteins in the diet consumed by each patient at the baseline. All patients were sufficiently adherent to the dietary treatment but, during dietary interviews, constantly declared to avoid dietary prescriptions 1–2 times/week and, in those cases, Phe intake was unquantifiable. All patients had been previously tested for BH4 responsiveness (48 h-loading test, as described in [33]) and resulted as not-responders, except patient #7, who refused the testing. The principles of good clinical practice (GCP) were adhered to throughout the study, in accordance with the Declaration of Helsinki (and its amendments) and the International Conference on Harmonization (ICH)/GCP guidelines. The study was performed in compliance with local regulatory requirements (EC Federico II University Hospital). Written informed consent was obtained from all participants or their legal representatives.

### 2.2. Study Protocol

**Figure 1:**
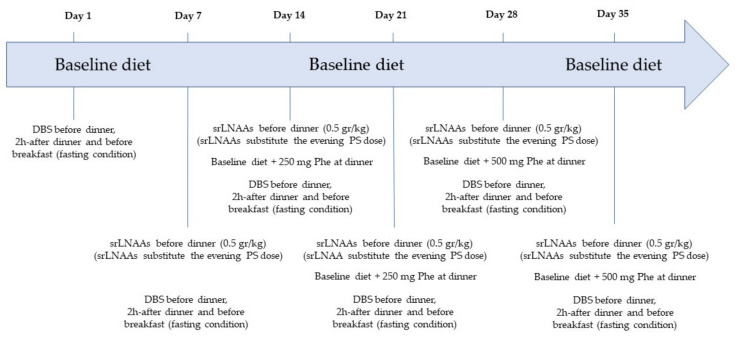
Study protocol.

The study protocol lasted a total of 6 weeks (Figure 1). Throughout the study, patients were asked to continue their usual dietary regimen and PS (baseline diet). On single and predefined days (days 1, 7, 14, 21, 28, and 35), patients were asked to collect dried blood spots (DBS) before dinner, 2h-after dinner, and the day after, before breakfast, in fasting conditions (>8 h) to assess the Phe and Tyr profile. On day 1 (baseline), patients did not change diet nor standard PS, did not take srLNAAs, and collected the DBS for Phe and Tyr profiles. On day 7, on their baseline diet, patients were asked to consume srLNAAs at the dose of 0.5 gr/kg before dinner in a single administration and to collect DBS for Phe and Tyr profiling. On days 14 and 21, patients were asked to consume srLNAAs (0.5 gr/kg before dinner), to add 250 mg of Phe to their usual diet at the evening meal, and to collect DBS for Phe and Tyr profiling. On days 28 and 35, patients were asked to consume srLNAAs (0.5 gr/kg before dinner), to add 500 mg of Phe to their usual diet at the evening meal, and to collect DBS for Phe and Tyr profiling. When taking the srLNAAs dose, patients were advised to avoid the evening consumption of their standard AA-formula. A meal diary was compiled by all patients and reviewed by two dietitians (AN and GE).

### 2.3. Dietary Phe Loads

Dietary Phe loads of 250 mg were performed at dinner on days 14 and 21 by adding 1 of the dietary products listed in Table 2 to the baseline diet. The 500 mg Phe loads were performed at dinner on days 28 and 35 by adding 2 of the products listed in Table 2.

### 2.4. SrLNAAs Supplementation

SrLNAAs (Neutrafenil Micro R^®^, PIAM Farmaceutici S.P.A., Genova, Italy) were prescribed at the dosage of 0.5 gr/kg 30 min before dinner in a single administration. The qualitative and quantitative composition of the product is described in Supplementary Table S1. The amino acids contained in the micro tablets are released within a time frame of 3 h after ingestion. In the case of overweight, the srLNAAs dose was calculated based on the patient’s ideal BMI (50° centile). On days 7, 14, 21, 28, and 35, patients took their standard PS only in the morning and at lunch, avoiding the dinner dose, which was substituted by srLNAAs.

### 2.5. Phe and Tyr Profiling

Phe, Tyr, and Phe/Tyr values were determined on DBS collected at home on days 1, 7, 14, 21, 28, and 35 at the following time points: before dinner, 2h-after dinner, and the following day before breakfast, in fasting conditions (>8 h). DBS were processed through LC-MS/MS analysis. Samples were prepared and analyzed as reported elsewhere [34].

### 2.6. Statistical Analysis

Data are expressed as means ± standard deviations (M ± SD). Medians (and ranges) were calculated for not normally distributed data sets. Plasma Phe, Tyr, and Phe/Tyr values collected at days 14 and 21 (250 mg Phe dietary load) and at days 28 and 35 (500 mg Phe dietary load) were pooled for statistical analysis to obtain the respective mean or median values. The normality of continuous data was assessed by the Shapiro–Wilks test. The Student’s *t*-test was used to compare means and the Mann–Whitney U-test to compare medians. Multiple comparisons between different protocol time points were carried out by the one-way ANOVA with repeated measures test followed by Tukey HSD (honestly significant difference) post-hoc test for normally distributed data, and by the Friedman test followed by the Wilcoxon signed-rank test for not normally distributed data. A two-tailed *p*-value < 0.05 was assumed as statistically significant. Statistical analysis was performed by SPSS 17.0 software.

## 3. Results

### 3.1. Plasma Phe Fluctuations at the Baseline, with and without srLNAAs, and during Phe Loads 

**Figure 2:**
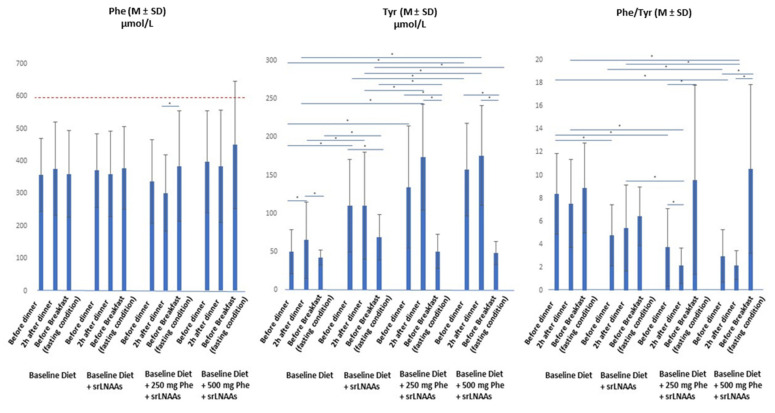
Mean plasma Phenylalanine, Tyrosine, and Phenylalanine/Tyrosine ratio (±SD) at the baseline, with and without srLNAAs, and during Phe loads. Multiple comparison analysis was carried out between the time points of the four dietary regimens. Statistically significant differences between different study time points are indicated by the blue lines; * *p* < 0.05. Plasma Phe, Tyr, and Phe/Tyr values collected at days 14 and 21 (250 mg Phe dietary load) and at days 28 and 35 (500 mg Phe dietary load) were pooled for statistical analysis to obtain the respective mean values. On days 7, 14, 21, 28, and 35, patients took their standard PS only in the morning and at lunch, avoiding the dinner dose, which was substituted by srLNAAs.

Table 3 shows the mean Phe profiles throughout the study (±SD). Figure 2 shows the comparisons between mean Phe values at each time point of the four dietary regimens and the statistical significances, available in detail in Supplementary Table S2. At the baseline (day 1), upon usual diet, mean Phe before dinner was 358 ± 113 µmol/L, 2h-after dinner was 376 ± 144 µmol/L, and before breakfast was 360 ± 134 µmol/L. The variations were not statistically significant. When a srLNAAs load (0.5 gr/kg) was added before dinner to the patients’ usual diet (day 7), mean Phe value before dinner was 371 ± 113 µmol/L, 2h-after dinner was 360 ± 131 µmol/L, and before breakfast was 378 ± 128 µmol/L. Also, in this case, Phe profiling did not show significant variations within the group or compared with the Phe values detected at the baseline (day 1). On days 14 and 21, each patient added to his usual diet a Phe load of 250 mg at dinner by choosing one of the products listed in Table 2; patients’ usual evening dose of PS was substituted by srLNAAs (0.5 gr/kg) 30 min before dinner. In this case, mean Phe before dinner was 337 ± 129 µmol/L, 2h-after dinner was 301 ± 117 µmol/L, and before breakfast was 384 ± 170 µmol/L (Table 3). The intragroup Phe analysis showed that Phe collected 2h-after dinner had a borderline significant reduction compared to Phe before dinner (*p*-value 0.05) and was significantly lower compared to mean Phe value before breakfast (*p*-value 0.04, Figure 2). No statistically significant difference was found between mean Phe values derived from a 250 mg Phe dietary load compared with baseline diets with or without srLNAAs (Figure 2, Supplementary Table S2). Finally, at days 28 and 35, each patient was asked to add to his usual diet a Phe load of 500 mg at dinner by choosing two of the products listed in Table 2; patients’ usual evening dose of PS was substituted by srLNAAs (0.5 gr/kg) 30 min before dinner. In this case, mean Phe value before dinner was 397 ± 157 µmol/L, 2h-after dinner was 384 ± 173 µmol/L, and before breakfast was 450 ± 196 µmol/L. Despite the mean plasma values being higher compared to the previous dietary regimens, the differences were not statistically significant, and the mean Phe remained below 600 µmol/L, according to the European guidelines [1]. The analysis of the Phe profiles for each patient showed that only 28.6% (4 patients; 3M/1F) exceeded the critical threshold of 600 µmol/L in one or more of the study time points, mostly after the overnight fast. Of these 4 patients, 2 (Pt #8 and Pt #13) presented plasma Phe values > 600 µmol/L at days 1 and 7, when no dietary Phe load was planned. In detail: Pt #6 had a Phe value of 644 and 631 µmol/L on day 28 (before dinner and 2h-after dinner) and of 765 µmol/L on day 35 (before breakfast); Pt #8 experienced a plasma Phe of 650 and 640 µmol/L 2h-after dinner on days 1 and 7, of 794 µmol/L before breakfast on day 28, and of 649 and 764 µmol/L before breakfast on day 28 and day 35; Pt #11 presented a Phe value of 615 µmol/L on day 28 (2h-after dinner), and of 677 and 660 µmol/L on day 35, 2h-after dinner and before breakfast, respectively; Pt #13 experienced a plasma Phe of 628 µmol/L before breakfast on day 1, 682 µmol/L before breakfast on day 7, 771 µmol/L before breakfast on day 14, 672 µmol/L before breakfast on day 21, 648 µmol/L before breakfast on day 28, and 640 µmol/L and 781 µmol/L 2h-after dinner and before breakfast on day 35, respectively. Overall, these results show that, with the exception of a few outliers, srLNAAs can prevent the increase of plasma Phe after an acute Phe dietary load.

### 3.2. Plasma Tyr Fluctuations at the Baseline, with and without srLNAAs, and during Phe Loads 

Table 3 shows mean Tyr profiles throughout the study. Figure 2 shows the comparisons between mean Tyr values at each time point of the four dietary regimens and the statistical significances, available in detail in Supplementary Table S3. At the baseline (day 1), patients’ mean Tyr profile on their usual diet showed a statistically significant increase 2h-after dinner compared with Tyr measured before dinner, as a consequence of the amount of Tyr derived from both natural proteins and the evening PS dose (*p*-value 0.01), and a subsequent significant reduction of plasma Tyr value in fasting conditions, before breakfast (Figure 2). At day 7 (baseline diet + srLNAAs), Tyr values significantly increased at all time points when compared with Tyr profiles registered at day 1 (Figure 2, Supplementary Table S3). At days 14 and 21, the mean Tyr registered before dinner and 2h-after dinner was significantly higher compared with the corresponding time points at the baseline (day 1) (Figure 2, Supplementary Table S3). Conversely, mean Tyr before breakfast, after the overnight fast, was significantly lower compared to the corresponding time point at day 7 but was not different compared to the baseline mean value (day 1). At days 28 and 35, when 500 mg Phe from natural proteins were added to the evening meal, mean Tyr profiles were comparable to those achieved at days 14 and 21 and were significantly higher than mean Tyr measured at day 1 and day 7, with the only exception of Tyr values collected after the overnight fast, which were significantly lower compared with the corresponding values of day 7 (baseline diet + srLNAAs), but not different from the mean values registered at the baseline (day 1) (Figure 2, Supplementary Table S3). Taken together, these results indicate that srLNAAs added to a protein-restricted diet are effective in increasing blood Tyr, in agreement with the previous data [27,29]; the effect is amplified by the addition of amounts of natural proteins to the diet.

### 3.3. Plasma Phe/Tyr Ratio at the Baseline, with and without srLNAAs, and During Phe Loads 

Phe/Tyr ratio was calculated at each time point. Mean or median values are shown in Table 3 and statistically significant data in Figure 2 and Supplementary Table S4. Compared with baseline measures, Phe/Tyr ratio profiling showed an overall significant reduction throughout the study with the addition of srLNAAs, with the exception of the breakfast values that were higher at days 14–21 and 28–35 compared with the mean ratios registered at days 1 and 7, despite the differences were not statistically significant.

### 3.4. Tolerability and Adverse Events

The srLNAAs load of 0.5 gr/kg before dinner was well tolerated in all subjects. Patients consumed the micro tablets in water or fruit juice. No adverse events were associated with srLNAAs. Hyperphenylalaninemia above the recommended upper reference range is itself an undesirable effect, and during the study, 4 patients experienced Phe values above 600 µmol/L at least in one of the study time points. However, in 2 out of 4 patients, Phe values were over 600 µmol/L at one or more time points of days 1 and 7 of the trial, where no oral Phe load was added.

## 4. Discussion

Following the diet for PKU is challenging for patients and their families. Adherence to treatment decreases with age, and metabolic control worsens in more than half of the subjects, with the unique exception of pregnant women with PKU [12,13,14,35,36]. From adolescence onwards, European guidelines advise maintaining blood Phe values below 600 µmol/L [1]. Even though this recommended cut-off is significantly higher than the reference range adopted in the US (<360 µmol/L in all age groups), blood Phe values within the safe range are difficult to maintain even for the most willing patients. Thanks to neonatal metabolic screening programs and early dietary intervention, neurological damage can be averted, and patients with PKU have the same school, social, and working opportunities as their peers. However, with increasing age, the complexity of social interactions and commitments requires flexibility that the actual dietary therapy does not allow. Proper compliance to the PKU diet requires patients to weigh portions of food, consume special low-protein foods, and consume PS doses divided throughout the day. As a result, the complexity of this program hinders everyday-life activities, and, in reality, patients with PKU do not have the same opportunities as their peers. Indeed, patients preferentially consume their meals at home, travel little, and feel obliged to select less dynamic working opportunities in order to be able to follow the diet. In this conflicting dichotomy between care for their health and social needs, even those patients committed to dietary therapy state that they have lunch or dinner out of the home 1–3 times/week, with a Phe intake 3–4 times higher than the prescribed quantity or unquantifiable [13,28]. The present study is devoted to the needs of this subgroup of patients. Based on the hypothesis that fluctuations of plasma Phe may hamper cognitive outcome as well as average high Phe levels [37,38,39], the study explores the effectiveness of srLNAAs to prevent plasma Phe peaks after acute oral Phe loads of 250 mg and 500 mg derived from natural protein-containing foods. The addition of these conspicuous Phe loads was intended to imitate (with regard to the quantity of Phe) a hypothetical meal that could be consumed at a bar or a pub. In real life, merely by way of example, the Phe load of 250 mg would correspond to a large portion (typically 200/250 gr) of fried potatoes or baked potatoes, or a small portion (typically gr 100/150) of fried potatoes + n.1 Wurstel (gr 25) or n.2 pizza mignon (5/7 cm diameter), such as those typically offered in bars as appetizers. The oral Phe load of 500 mg would correspond to a tomato or bell pepper baby pizza (150 gr) or a large portion (200/250 gr) of fried potatoes or baked potatoes + n.2 Wurstels (gr 50). As graphically shown in Figure 2, this group of patients presented mean plasma Phe values below 600 µmol/L on their baseline therapy consisting of a low-protein diet, PS, and special low-protein food. The addition of srLNAAs before the evening meal did not change plasma Phe values on the baseline diet. Surprisingly, the addition to the evening meal of a conspicuous amount of 250 mg and 500 mg of Phe derived from natural proteins did not induce a significantly different rise of mean Phe levels compared with the baseline profile, and mean Phe values remained below the recommended upper plasma Phe value of 600 µmol/L, with the exception of a few outlier values. This observation could be due to the greater physiological absorption of srLNAAs over a 3h-period and the consequent more efficient competition at the intestinal LAT2 transporter. In addition, the greater physiological amino acid absorption may stimulate amino acid retention and anabolism [40]. SrLNAAs may also reduce or prevent nocturnal catabolism. The srLNAAs supplementation to the baseline dietary regimen induced a significant rise in Tyr blood levels, confirming previous observations [27,29]. Mean Tyr values showed a further increase when the oral Phe loads of 250 mg and 500 mg were performed at dinner. The significant reduction of plasma Tyr after the overnight fast at days 14–21 and 28–35 compared with day 7 could be a consequence of improved anabolism. Therefore, the administration of srLNAAs prevents fluctuations in plasma Phe and provides increased amounts of Tyr disposable for protein synthesis and for the putative competition with the brain LAT1 transporter. In this study, we decided to avoid the Phe loads without the simultaneous assumption of srLNAAs. This could be a limitation of the study since we have no data on Phe peaks in plasma during Phe dietary loads without the investigational product. However, the proposed amount of dietary Phe was, in a single meal, 2–3-times higher than the patients’ tolerance. Since the intestinal absorption of the standard PS is very rapid and there is little amino acid retention, we felt that those dietary Phe increases would put patients at risk of high plasma Phe values without the simultaneous addition of srLNAAs. Indeed, 86% of the patients had severe PAH mutations with null or very low predicted enzyme activity and poor capability of Phe clearance. During the late 1970s and 1980s, Phe-loading tests with high Phe oral doses were conducted in infant dietary treated PKU patients to discriminate between classic or variant PKU, inducing picks up to 2000 µmol/L of plasma Phe followed by slow plasma Phe clearance after 48–72 h [41,42,43]. Assuming that the more physiological absorption of srLNAAs could minimize the increase in plasma Phe, particularly in the morning, we considered it safer to perform dietary Phe increases only in combination with srLNAAs. 

## 5. Conclusions

In conclusion, data from this study show that srLNAAs may be beneficial to avoid plasma Phe increase after acute oral Phe loads with natural protein-containing food and may putatively protect brain neurotransmitter homeostasis by increasing Tyr and other LNAAs, as hypothesized in previous studies and recently reviewed [44]. In selected patients, the use of srLNAAs may greatly improve metabolic control and, if it is true that fluctuations in Phe levels affect the cognitive outcome [38,39], srLNAAs may ameliorate health and long term outcomes of adolescents and adult patients with PKU. We speculate that in specific circumstances in which selected patients foresee an increase of dietary Phe intake, single srLNAAs administrations could be used upon medical advice to stabilize blood Phe values. SrLNAAs may also be useful in the daily long term dietary management of PKU patients treated with diet alone or in combination with sapropterin. Physicians should become increasingly engaged in the development of personalized therapies tailored to patients’ needs with the aim of creating the best conditions for each subject to achieve adequate metabolic control and improve their long-term prognosis. While new pharmacological therapies are being introduced into clinical practice, such as enzyme substitution and gene therapies, dietary treatment should become dynamic and flexible according to patients’ needs.

## Figures and Tables

**Table 1:** Patients’ demographics, phenotype, genotype and dietary amino acid intake.

Patient #	SEX (M/F)	Age (years)	PHE at Diagnosis (µmol/L)	Tolerance (mg/day)	PAH Mutations (Allele 1/Allele 2)	AA from PS (gr)	AA from Natural Protein (gr)	srLNAAs Load (gr)
1	F	28	1500	350	R261Q/IVS10nt-11G>A	40	15	30
2	F	33	660	500	L48S/R158Q	15	37	35
3	F	29	1450	250	1055delG/1055delG	44.5	9.5	30
4	F	24	1300	280	R252W/R408W	54	10	30
5	F	30	1900	350	R261Q/IVS07nt3G>C	52	18	35
6	M	22	1100	390	IVS10nt-11G>A/116-118delTCT	52	21	30
7	M	18	620	500	I94S/I94S	56	30	35
8	M	21	1570	325	pS16XFsx1/y343C+[Ivs3-22C>T/Q232Q]	50	15	36
9	F	20	2070	225	R158Q/R158Q	42	9	26
10	F	17	1530	211	IVS10nt-11G>A/pF55LFs	37.5	10	26
11	M	17	1028	370	L48S/IVS10nt-11G>A	37.5	9	33
12	F	21	2180	310	IVS10nt-11G>A/R261Q	37.5	15	26
13	F	14	883	355	n.a.	37.8	9.4	22
14	F	14	1936	280	IVS10nt-11G>A/IVS10nt-11G>A	40	9.5	22

M: male; F: female; Y: years; Phe: phenylalanine; PAH: phenylalanine hydroxylase; AA: amino acids; PS: protein substitutes; srLNAAs: slow-release large neutral amino acids, #: patient number.

**Table 2:** List of the dietary products and quantities (in grams) equivalent to 250 mg Phe. Patients were asked to choose 1 product on the list to add 250 mg Phe and 2 products to add 500 mg Phe.

Food Product	Quantity of Product (gr)	Content of Natural Proteins (gr)
Potatoes	280	5.8
Fish stick	50	5.5
Sliced cheese	20	4.9
Baked ham	30	5.9
Spreadable cheese wedges	40	4.4

**Table 3:** Mean values (±SD) of plasma Phenylalanine, Tyrosine, and Phenylalanine/Tyrosine ratio.

Diet	Time-Point	Phe	Tyr	Phe/Tyr
Baseline Diet (Day 1)	Before dinner	358 ± 113	50 ± 29	8 ± 3
	2h-after dinner	376 ± 144	65 ± 50	7 ± 4
	Before breakfast (Fasting condition)	360 ± 134	42 ± 10	9 ± 4
Baseline diet + srLNAAs (Day 7)	Before dinner	371 ± 113	110 ± 60 *	5 ± 2 *
	2h-after dinner	360 ± 131	110 ± 70 *	5 ± 4
	Before breakfast (Fasting condition)	378 ± 128	69 ± 30 *	6 ± 3
Baseline diet + 250 mg Phe + srLNAAs (Day 14, 21)	Before dinner	337 ± 129	134 ± 80 *	4 ± 3 *
	2h-after dinner	301 ± 117	174 ± 69 *	2 ± 2 *
	Before breakfast (Fasting condition)	384 ± 170	50 ± 22	9 ± 8 6 (2–29)
Baseline diet + 500 mg Phe + srLNAAs (Day 28, 35)	Before dinner	397 ± 157	157 ± 61 *	3 ± 2 *
	2h-after dinner	384 ± 173	176 ± 65 *	2 ± 1 *
	Before breakfast (Fasting condition)	450 ± 196	48 ± 15	10 ± 7 8 (4–30)

Phe and Tyr values are expressed in µmol/L. Data are presented as means ± SD. In the case of not normally distributed data set, median values and range are also reported [Median (range)]. Plasma Phe, Tyr, and Phe/Tyr values collected at days 14 and 21 (250 mg Phe dietary load) and at days 28 and 35 (500 mg Phe dietary load) were pooled for statistical analysis to obtain the respective mean or median values. The statistically significant different time point values of the three experimental diets compared with the respective baseline time points are reported in the table, marked with an asterisk (*).

## Data Availability

The data presented in this study are available on request from the corresponding author.

## Supplementary Materials

The following are available online at https://www.mdpi.com/article/10.3390/nu13114012/s1. Table S1. Qualitative and quantitative composition per 100 g of the slow-release large neutral amino acids formulation (Neutrafenil Micro R®, PIAM Farmaceutici S.P.A., Genova Italy) Table S2. Statistical significance (*p*-values) of comparisons between mean Phe values at each timepoint of the four dietary regimens. Table S3. Statistical significance (*p*-values) of comparisons between mean Tyr values at each time point of the four dietary regimens. Table S4. Statistical significance (*p*-values) of comparisons between Phe/Tyr values at each time point of the four dietary regimens.
