# Supplementary material for: Beneficial Effects of Slow-Release Large Neutral Amino Acids after a Phenylalanine Oral Load in Patients with Phenylketonuria
Source: Nutrients. 2021 Nov 10;13(11):4012. doi: 10.3390/nu13114012 (PMC8618154; doi:10.3390/nu13114012)
Supplement: Supplementary file 1 [file nutrients-13-04012-s001.zip › Nutrients Supplementary Tables second revision/Supplementary table 1 (2) (2).pdf]

Supplementary Table S1. Qualitative and quantitative composition per 100 g of the slow-release large neutral amino acids formulation (Neutrafenil Micro R®, PIAM Farmaceutici S.P.A.,Genova, Italy).

Energy	1686 kj<br>399 Kcal
Fat<br>of which saturated fatty acids	5.3 g<br>5.3 g
Carbohydrates<br>of which sugar	12.0 g<br>0
Fibers	5.8 g
Protein Equivalent	70.73 g
Salt	1.6 g
L-arginine	1.92 g
Aspartate	4.95 g
L-phenylalanine	0 g
L-isoleucine	10.0 g
L-histidine	3.36 g
L-leucine	12.0 g
L-lysine	5.44 g
L-methionine	2.72 g
L-tyrosine	24 g
L-threonine	2.56 g
L-tryptophan	8.0 g
L-valine	10.0 g
