# Supplementary material for: Beneficial Effects of Slow-Release Large Neutral Amino Acids after a Phenylalanine Oral Load in Patients with Phenylketonuria
Source: Nutrients. 2021 Nov 10;13(11):4012. doi: 10.3390/nu13114012 (PMC8618154; doi:10.3390/nu13114012)
Supplement: Supplementary file 1 [file nutrients-13-04012-s001.zip › Nutrients Supplementary Tables second revision/Supplementary table 2 (2) (1).pdf]

Supplementary table S2. Statistical significance (p-values) of comparisons between mean Phe values at each time-point of the four dietary regimens. Data represent p-values.

			Baseline Diet			Baseline diet + srLNAAs			Baseline diet + 250 mg Phe + srLNAAs			Baseline diet + 500 mg Phe + srLNAAs		
	Time-points		1	2	3	4	5	6	7	8	9	10	11	12
			Before dinner	2h-after dinner	Before breakfast	Before dinner	2h-after dinner	Before breakfast	Before dinner	2h-after dinner	Before breakfast	Before dinner	2h-after dinner	Before breakfast
Baseline Diet	1	Before dinner		0.7	0.9	0.8			0.6			0.4		
	2	2h-after dinner	0.7		0.7		0.7			0.07			0.9	
	3	Before breakfast	0.9	0.7				0.7			0.6			0.1
Baseline diet + srLNAAs	4	Before dinner	0.8				0.8	0.9	0.4			0.6		
	5	2h-after dinner		0.7		0.8		0.7		0.1			0.6	
	6	Before breakfast			0.7	0.9	0.7				0.9			0.2
Baseline diet + 250 mg Phe + srLNAAs	7	Before dinner	0.6			0.4				0.3	0.2	0.12		
	8	2h-after dinner		0.07			0.1		0.3		**0.04**		0.09	
	9	Before breakfast			0.6			0.9	0.2	**0.04**				0.2
Baseline diet + 500 mg Phe + srLNAAs	10	Before dinner	0.4			0.6			0.12				0.7	0.3
	11	2h-after dinner		0.9			0.6			0.09		0.7		0.2
	12	Before breakfast			0.1			0.2			0.2	0.3	0.2	

For each dietary regimen, comparisons were done between all time-points (i.e: 2 vs 1; 3 vs 1, 3 vs 2; 6 vs 4, 6 vs 4, 6 vs 5, etc.); among different dietary regimens, comparisons were performed between the same time-point (i.e: 4 vs 1, 5 vs 2, 6 vs 3, 7 vs 1, 8 vs 2, 9 vs 3, etc.). Multiple comparisons between different protocol time-points were carried out by one-way ANOVA followed by Tukey HSD post-hoc test for normally distributed data.
