# Supplementary material for: Beneficial Effects of Slow-Release Large Neutral Amino Acids after a Phenylalanine Oral Load in Patients with Phenylketonuria
Source: Nutrients. 2021 Nov 10;13(11):4012. doi: 10.3390/nu13114012 (PMC8618154; doi:10.3390/nu13114012)
Supplement: Supplementary file 1 [file nutrients-13-04012-s001.zip › Nutrients Supplementary Tables second revision/Supplementary table 3 (2) (2).pdf]

Supplementary table S3. Statistical significance (p-values) of comparisons between mean Tyr values at each time-point of the four dietary regimens. Data represent p-values.

			Baseline Diet			Baseline diet + srLNAA			Baseline diet + 250 mg Phe + srLNAA			Baseline diet + 500 mg Phe + srLNAA		
	Time-points		1	2	3	4	5	6	7	8	9	10	11	12
			Before dinner	2h-after dinner	Before breakfast	Before dinner	2h-after dinner	Before breakfast	Before dinner	2h-after dinner	Before breakfast	Before dinner	2h-after dinner	Before breakfast
Baseline Diet	1	Before dinner		**0.01**	0.7	**0.002**			**0.001**			**<0.0001**		
	2	2h-after dinner	**0.01**		**0.01**		**0.01**			**<0.0001**			**<0.0001**	
	3	Before breakfast	0.7	**0.01**				**0.0008**			0.2			0.19
Baseline diet + srLNAA	4	Before dinner	**0.002**				0.69	**0.02**	0.29			**0.02**		
	5	2h-after dinner		**0.01**		0.69		0.07		**0.01**			**0.004**	
	6	Before breakfast			**0.0008**	**0.02**	0.07				**0.03**			**0.004**
Baseline diet + 250 mg Phe + srLNAA	7	Before dinner	**0.001**			0.29				0.05	**<0.0001**	0.3		
	8	2h-after dinner		**<0.0001**			**0.01**		0.05		**<0.0001**		0.9	
	9	Before breakfast			0.2			**0.03**	**<0.0001**	**<0.0001**				0.65
Baseline diet + 500 mg Phe + srLNAA	10	Before dinner	**<0.0001**			**0.02**			0.3				0.2	**<0.0001**
	11	2h-after dinner		**<0.0001**			**0.004**			0.9		0.2		**<0.0001**
	12	Before breakfast			0.19			**0.004**			0.65	**<0.0001**	**<0.0001**	

For each dietary regimen, comparisons were done between all time-points (example: 2 vs 1; 3 vs 1, 3 vs 2; 6 vs 4, 6 vs 4, 6 vs 5, etc.); among different dietary regimens, comparisons were performed between the same time-point (example: 4 vs 1, 5 vs 2, 6 vs 3, 7 vs 1, 8 vs 2, 9 vs 3, etc.). Multiple comparisons between different protocol time-points were carried out by one-way ANOVA followed by Tukey HSD post-hoc test for normally distributed data.
