# Supplementary material for: Beneficial Effects of Slow-Release Large Neutral Amino Acids after a Phenylalanine Oral Load in Patients with Phenylketonuria
Source: Nutrients. 2021 Nov 10;13(11):4012. doi: 10.3390/nu13114012 (PMC8618154; doi:10.3390/nu13114012)
Supplement: Supplementary file 1 [file nutrients-13-04012-s001.zip › Nutrients Supplementary Tables second revision/Supplementary Table 4 _second revision.pdf]

Supplementary Table S4. Statistical significance (p-values) of comparisons between Phe/Tyr values at each time-point of the four dietary regimens. Data represent p-values.

			Baseline Diet			Baseline diet + srLNAAs			Baseline diet + 250 mg Phe + srLNAAs			Baseline diet + 500 mg Phe + srLNAAs		
	Time-points		1	2	3	4	5	6	7	8	9	10	11	12
			Before dinner	2h-after dinner	Before breakfast	Before dinner	2h-after dinner	Before breakfast	Before dinner	2h-after dinner	Before breakfast	Before dinner	2h-after dinner	Before breakfast
Baseline Diet	1	Before dinner		0.5	0.5	**0.005**			**0.001**			**<0.00001**		
	2	2h-after dinner	0.5		0.5		0.1			**<0.00001**			**<0.00001**	
	3	Before breakfast	0.5	0.5				0.06			0.7			0.4
Baseline diet + srLNAAs	4	Before dinner	**0.005**				0.6	0.1	0.4			**0.03**		
	5	2h-after dinner		0.1		0.6		0.4		**0.0002**			**0.0001**	
	6	Before breakfast			0.06	0.1	0.4				0.2			0.05
Baseline diet + 250 mg Phe + srLNAAs	7	Before dinner	**0.001**			0.4				0.08	**0.001**	0.4		
	8	2h-after dinner		**<0.00001**			**0.0002**		0.08		**0.00001**		0.9	
	9	Before breakfast			0.7			0.2	**0.001**	**0.00001**				0.6
Baseline diet + 500 mg Phe + srLNAAs	10	Before dinner	**<0.00001**			**0.03**			0.4				0.1	**<0.00001**
	11	2h-after dinner		**<0.00001**			**0.0001**			0.9		0.1		**<0.00001**
	12	Before breakfast			0.4			0.05			0.6	**<0.00001**	**<0.00001**	

For each dietary regimen, comparisons were done between all time-points (i.e: 2 vs 1; 3 vs 1, 3 vs 2; 6 vs 4, 6 vs 4, 6 vs 5, etc.); among different dietary regimens, comparisons were performed between the same time-point (i.e: 4 vs 1, 5 vs 2, 6 vs 3, 7 vs 1, 8 vs 2, 9 vs 3, etc.). Multiple comparisons between different protocol time-points were carried out by the one-way ANOVA with repeated measures test followed by Tukey HSD (honestly significant difference) post-hoc test for normally distributed data, and by the Friedman test followed by the Wilcoxon signed-rank test for not normally distributed data.
